# The Japanese Herbal Medicine Hangeshashinto Induces Oral Keratinocyte Migration by Mediating the Expression of CXCL12 Through the Activation of Extracellular Signal-Regulated Kinase

**DOI:** 10.3389/fphar.2021.695039

**Published:** 2022-01-18

**Authors:** Kanako Miyano, Seiya Hasegawa, Noriho Asai, Miaki Uzu, Wakako Yatsuoka, Takao Ueno, Miki Nonaka, Hideaki Fujii, Yasuhito Uezono

**Affiliations:** ^1^ Division of Cancer Pathophysiology, National Cancer Research Institute, Tokyo, Japan; ^2^ Department of Pain Control Research, The Jikei University School of Medicine, Tokyo, Japan; ^3^ Laboratory of Medicinal Chemistry, School of Pharmacy, Kitasato University, Tokyo, Japan; ^4^ Vitrigel Project Research Team, Institute of Agrobiological Sciences, National Agriculture and Food Research Organization, Tsukuba, Japan; ^5^ Dental Division, National Cancer Center Hospital, 5-1-1, Tsukiji, Japan; ^6^ Supportive and Palliative Care Research Support Office, National Cancer Center Hospital East, Kashiwa, Japan

**Keywords:** hangeshashinto, oral ulcerative mucositis, oral keratinocytes, CXCL12, extracellular signal-regulated kinase

## Abstract

Several clinical studies have reported that Japanese herbal medicine Hangeshashinto (HST) has beneficial effects on chemotherapy-induced oral ulcerative mucositis (OUM). Our previous research demonstrated that HST improves chemotherapy-induced OUM through human oral keratinocyte (HOK) migration, which was suppressed by mitogen-activated protein kinase (MAPK) and C-X-C chemokine receptor 4 (CXCR4) inhibitors. However, the association between these molecules and HOK migration was unclear. Here, we examined the effects of HST on the expression of CXCR4/CXCR7 and C-X-C motif chemokine ligands 11 and 12 (CXCL11/CXCL12) in HOKs. Our results indicated that HST upregulated CXCL12, but not CXCR4, CXCR7, nor CXCL11 in HOKs. HST-induced expression of CXCL12 was significantly suppressed by an inhibitor of extracellular signal-regulated kinase (ERK), but not of p38 and c-Jun N-terminal kinase (JNK). In addition, HST induced phosphorylation of ERK in HOKs. These findings suggest that HST enhances HOK migration by upregulating CXCL12 via ERK.

## 1 Introduction

Cancer patients receiving chemotherapy, radiotherapy, hematopoietic stem cell transplant, or terminal care often experience severe oral ulcerative mucositis (OUM), which evokes painful inflammation and limits their basic day-to day activities, such as “eating, drinking, and talking” (McGuire et al., 1993; Sonis, 1998; Dodd et al., 2000; Dörr et al., 2002; Sonis, 2004; Duncan et al., 2005; Jones et al., 2006; Vera-Llonch et al., 2006; Barber et al., 2007; El-Housseiny et al., 2007; Vera-Llonch et al., 2007; Bensinger et al., 2008; Sonis, 2010a; Sonis, 2010b; Miyano et al., 2016; Miyano et al., 2020). Additionally, OUM increases the risk of systemic infection via opportunistic microorganisms, which may lead to extension of hospitalization (Elting et al., 2003; Sonis, 2004; Elting et al., 2007; Yeoh et al., 2007; Lalla et al., 2014; Miyano et al., 2016). Further, OUM often forces patients with cancer to discontinue or modify their therapy regimen, which adversely affects their prognosis (Elting et al., 2003; Trotti et al., 2003; Sonis, 2010a; Miyano et al., 2016). Therefore, effective management of OUM is indispensable for improving both patient quality of life and prognosis (Miyano et al., 2016).

Although chemotherapy-induced OUM is associated with the use of various anti-cancer drugs, there are not many effective prevention methods or therapeutic modalities (Miyano et al., 2016). Hangeshashinto (HST), a traditional Japanese medicine (Kampo medicine) that contains extracts of seven botanical drugs, was approved by Japan’s Ministry of Health, Labour and Welfare as a prescription treatment for OUM. From the 16th century to the present, HST has been used in Japan to treat inflammatory diarrhea, gastritis, and oral mucositis (Uezono et al., 2012; Miyano et al., 2016). A recent double-blind, placebo-controlled, randomized study reported that the repetitive use of HST-containing mouthwash effectively improved chemotherapy-induced OUM in patients with colorectal cancer or gastric cancer (Matsuda et al., 2015). Basic research indicated that HST enhanced OUM healing through multiple pharmacological actions, such as anti-oxidant, anti-inflammatory, anti-bacterial, and analgesic activities (Fukamachi et al., 2015; Matsumoto et al., 2015; Hiroshima et al., 2016; Hitomi et al., 2016; Hitomi et al., 2017). With regard to anti-inflammatory effects, we previously determined that various ingredients in HST decrease interleukin 1β-induced prostaglandin E2 (PGE2) production in human oral keratinocytes (HOKs) with multi-targeting effects, such as dual suppression of cyclooxygenase-2 expression and PGE2 metabolic activity (Kono et al., 2014). Moreover, our recent *in vitro* and *in vivo* studies revealed that HST directly affects OUM and enhances tissue repair through migration of HOKs, involving activation of mitogen-activated protein kinases (MAPKs), including extracellular-signal-regulated kinase (ERK), p38, and c-Jun N-terminal kinase (JNK), and C-X-C chemokine receptor 4 (CXCR4) (Miyano et al., 2020). In the present study, we investigated the effects of HST on the expression of endogenous CXCR4 agonists (C-X-C chemokine ligands CXCL11 and CXCL12) and the receptors CXCR4 and CXCR7 to clarify how MAPKs and CXCR4 induce HOK migration. We analyzed the effects of several MAPK inhibitors on the expression of CXCL12, and also examined the effects of HST treatment on MAPK phosphorylation in migrating HOKs.

## 2 Materials and Methods

### 2.1 Chemicals and Reagents

The following reagents were used: fetal bovine serum (FBS) and Keratinocyte-Serum Free Medium (SFM) (1X) (Gibco, Carlsbad, CA, United States); trypsin and trypsin neutralizing solution (TNS; Lonza, Basel, Switzerland); penicillin/streptomycin, dimethyl sulphoxide (DMSO), and U0126 (Nacalai Tesque, Kyoto, Japan); poly-L-lysine (PLL), SB202190 (Sigma-Aldrich, St. Louis, MO, United States); Cellmatrix^®^ I-P (Nitta Gelatin Inc., Osaka, Japan); phosphate-buffered saline (PBS; Nissui Pharmaceutical Co., Osaka, Japan); BDPA-Zn (Fujifilm Wako Pure Chemical, Osaka, Japan); and JNK inhibitor II (Calbiochem, San Diego, CA, United States).

HST extract powder (Lot No. 2180014010), the base powder without excipients, was obtained from Tsumura & Co. (Ibaraki, Japan), manufactured as an aqueous extract mixture of seven botanical drugs. All items prescribed in Hangeshashinto are listed as in the Japanese Pharmacopoeia: *Pinellia tuber* (5.0 g, tuber of *Pinellia ternate* (Thunb.) Makino (Araceae)), *Scutellaria root* (2.5 g, root of *Scutellaria baicalensis* Georgi (Lamiaceae)), *Processed ginger* (2.5 g, rhizome of *Zingiber officinale* Roscoe (Zingiberaceae)), *Glycyrrhizae Radix* (2.5 g, root of *Glycyrrhiza uralensis* Fisch. ex DC. (Fabaceae) or *Glycyrrhiza glabra* L. (Fabaceae)), *Ziziphi Fructus* (2.5 g, fruit of *Ziziphus jujuba* Mill. (Rhamnaceae)), *Ginseng Radix* (2.5 g, root of *Panax ginseng* C. A. Mey. (Araliaceae)), and *Coptis rhizome* (1.0 g, rhizome of *Coptis japonica* (Thunb.) Makino (Ranunculaceae), *Coptis chinensis* Franch. (Ranunculaceae), *Coptis deltoidei* C. Y. Cheng and P. K. Hsiao (Ranunculaceae) or *Coptis teeta* Wall. (Ranunculaceae)). Briefly, the mixture of the seven raw materials was extracted in boiling water for 1 h, and the extract was then separated from insoluble waste. The separated extract was concentrated under reduced pressure and then spray-dried to produce the extract powder of HST. The yield of the extract was about 24.3%. The three-dimensional high-performance liquid chromatograph (3D-HPLC) profile of HST was created by Tsumura & Co., showing at Supplementary Figure S1. For the analysis of components, the dried extract (1.0 g) of HST was extracted with methanol (20 ml) under ultrasonication for 30 min and was centrifuged at 3,000 rpm for 5 min. The supernatants were filtered with a membrane filter (0.45 μm) and then submitted for HPLC analysis (30 μL). HPLC apparatus consisted of a Shimadzu LC 10A (analysis system software: CLASS-M10A ver. 1.64, Tokyo, Japan) equipped with a multiple wavelength detector (UV 200–400 nm) (Shimadzu SPD-M10Avp, diode array detector), an auto injector (Shimadzu CTO-10AC). HPLC conditions were described as follows: column, ODS (TSK-GEL 80TS, 250 × 4.6 mm i.d., TOSOH, Tokyo, Japan); eluent, (A) 0.05M AcONH4 (pH 3.6) (B) 100% CH3CN. A linear gradient of 90% of A and 10% of B changing over 60 min to 0% A and 100% B was used. (And 100% B was continued for 20 min); temperature, 40°C; flow rate, 1.0 ml/min. The quality of HST was confirmed to fulfill the standard of the Japanese Pharmacopoeia. Specifically, the following marker compounds were included in the extract within the parenthesized rages: baicalin (70–210 mg), glycyrrhizic acid (22–66 mg), and berberine (7–21 mg). All voucher specimens of raw materials used were deposited in the herbarium of Tsumura & Co., with batch numbers (Supplementary Table S1).

HST extract powder was suspended in DMSO at 100 mg/ml, diluted 100 fold with culture medium, and filtered through a 0.45 μm membrane (ADVANTEC, Tokyo, Japan) to give a final concentration of 100 μg/ml.

### 2.2 Cell Culture

Primary HOKs (ScienCell Research Laboratories, Carlsbad, CA, United States) were cultured on poly-L-lysine-coated dishes in Keratinocyte-SFM (1X) supplemented with 10% FBS and penicillin (100 U/mL).

### 2.3 Scratch-Induced Migration Assay

HOK migration was evaluated using the IncuCyte ZOOM^®^ system (ESSEN BioScience, Ann Arbor, MI, United States), which enables real-time and quantitative live-cell analysis, as previously described (Miyano et al., 2020). HOKs were seeded at a concentration of 3.0 × 10^4^ cells/0.1 ml/well onto a 96-well ImageLock microplate (ESSEN BioScience), coated with 300 μg/ml Cellmatrix^Ⓡ^ I-P (Nitta Gelatin Inc., Osaka, Japan). The following day, the cells were scratched using a 96-well WoundMaker (ESSEN BioScience), and the culture medium was changed to assay medium (Keratinocyte-SFM (1X) containing 2% FBS). The cells were then treated with HST (100 μg/ml) and visually monitored every 2 h for 72 h. The area occupied by HOKs on the scratched area was quantified using IncuCyte™ scratch wound cell migration software (ESSEN BioScience).

### 2.4 Real-Time Quantitative PCR

HOKs were seeded at 6.5 × 10^5^ cells/2 ml/well onto a 6-well microplate (Thermo Fisher Scientific, Inc., Waltham, MA, United States), coated with 300 μg/ml Cellmatrix^Ⓡ^ I-P. The following day, the cells were scratched with a 1 ml syringe (Terumo Corporation, Tokyo, Japan) and the culture medium was changed to assay medium. The cells were then treated with HST for 48 h. Total RNA was extracted using the AllPrep DNA/RNA/Protein Mini Kit (QIAGEN, Hilden, Germany). Sample RNA (1.5 µg) was reverse-transcribed using the High-Capacity RNA-to-cDNA™ Kit (Thermo Fisher Scientific), according to the manufacturer’s instructions. Real-time quantitative PCR (RT-qPCR) analysis was conducted using LightCycler FastStart DNA Master PLUS SYBR Green I (Roche, Basel, Switzerland) on a LightCycler 2.0 system (Roche). Thermal cycling was initiated at 95°C for 1 min, followed by 50 cycles of 20 s at 95°C, 10 s at 58°C, and 10 s at 72°C. The glyceraldehyde-3-phosphate dehydrogenase (*GAPDH*) gene was used as a reference gene to normalize expression levels in RT-qPCR analysis. The primer sequences of CXCR4, CXCR7, CXCL12, and GAPDH are listed in Table 1. The PCR products were analyzed on 1.5% agarose gel and had the sizes expected from the known cDNA sequences. CXCL11 primers were purchased from Sino Biological (Beijing, China). RNA quantities of target genes were calculated using the Ct method (Livak and Schmittgen, 2001).

**TABLE 1:** Primer sequences of human CXCR4, CXCR7, CXCL12, and GAPDH.

	Forward primers (5′→3′)	Reverse primers (3′→5′)
CXCR4	CGT​CTC​AGT​GCC​CTT​TTG​TTC	CTG​AAG​TAG​TGG​GCT​AAG​GGC
CXCR7	CTA​TGA​CAC​GCA​CTG​CTA​CAT​C	CTG​TAC​GAG​ACT​GAC​CAC​C
CXCL12	ACA​CTC​CAA​ACT​GTG​CCC​TT	CTG​TAA​GGG​TTC​CTC​AGG​CG
GAPDH	GCT​CTC​TGC​TCC​TCC​TGT​TC	ACG​ACC​AAA​TCC​GTT​GAC​TC

### 2.5 Western Blotting

Sample proteins were extracted using the AllPrep DNA/RNA/Protein Mini Kit and diluted in sodium dodecyl sulfate (SDS) sample buffer (Nacalai Tesque). After heating for 5 min at 95°C, equal amounts of proteins were separated by SDS-polyacrylamide gel electrophoresis and blotted onto polyvinylidene difluoride (PVDF) membranes. The membranes were blocked with Blocking One solution (Nacalai Tesque) for 1 h at room temperature, and incubated overnight at 4°C with primary rabbit IgG antibodies against ERK1/2 (1:1,000; Cell Signaling Technology Inc., Danvers, MA, United States) and primary rabbit IgG antibodies against phospho-ERK1/2 (1:1,000; Cell Signaling Technology, Inc.). After washing, the membranes were further incubated with horseradish peroxidase-linked anti-rabbit IgG antibody (1:2,000; Cell Signaling Technology Inc.) for 2 h at room temperature. Immunoreactivity was detected using the Western Lightning ECL Pro system (Perkin Elmer Co., Ltd., Waltham, MA, United States). Finally, the band densities of both pERK and ERK were measured using ImageJ software (National Institutes of Health, Bethesda, MD, United States). ERK expression was calculated using the ratio of the phospho-ERK-specific band density/ERK-specific band density.

### 2.6 Statistical Analysis

**FIGURE 1:**
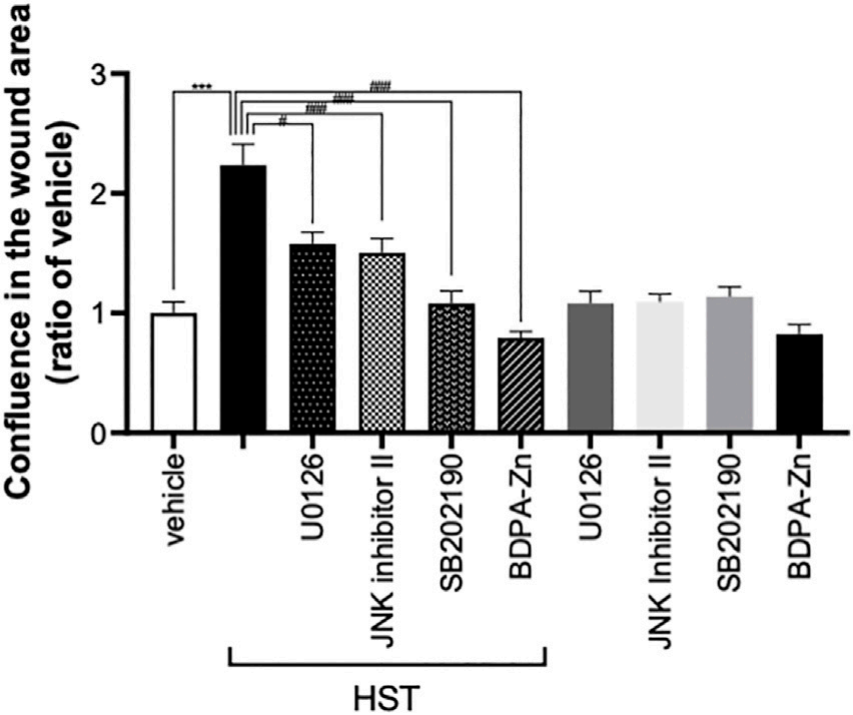
Effect of MAPK inhibitors on Hangeshashinto (HST)-induced migration of human oral keratinocyte (HOKs). HOKs were scratched and co-treated with HST, CXCR4 inhibitor BDPA-Zn, ERK inhibitor U0126, JNK inhibitor II, or p38 inhibitor SB202190 for 72 h. Data are expressed as the mean ± SEM (bars, *n* = 12–50). *** indicates *p* < 0.001, compared with vehicle; #, ##, #### indicates *p* < 0.05, *p* < 0.01, *p* < 0.001 compared with HST alone. Bonferroni’s comparison test following ANOVA.

**FIGURE 2:**
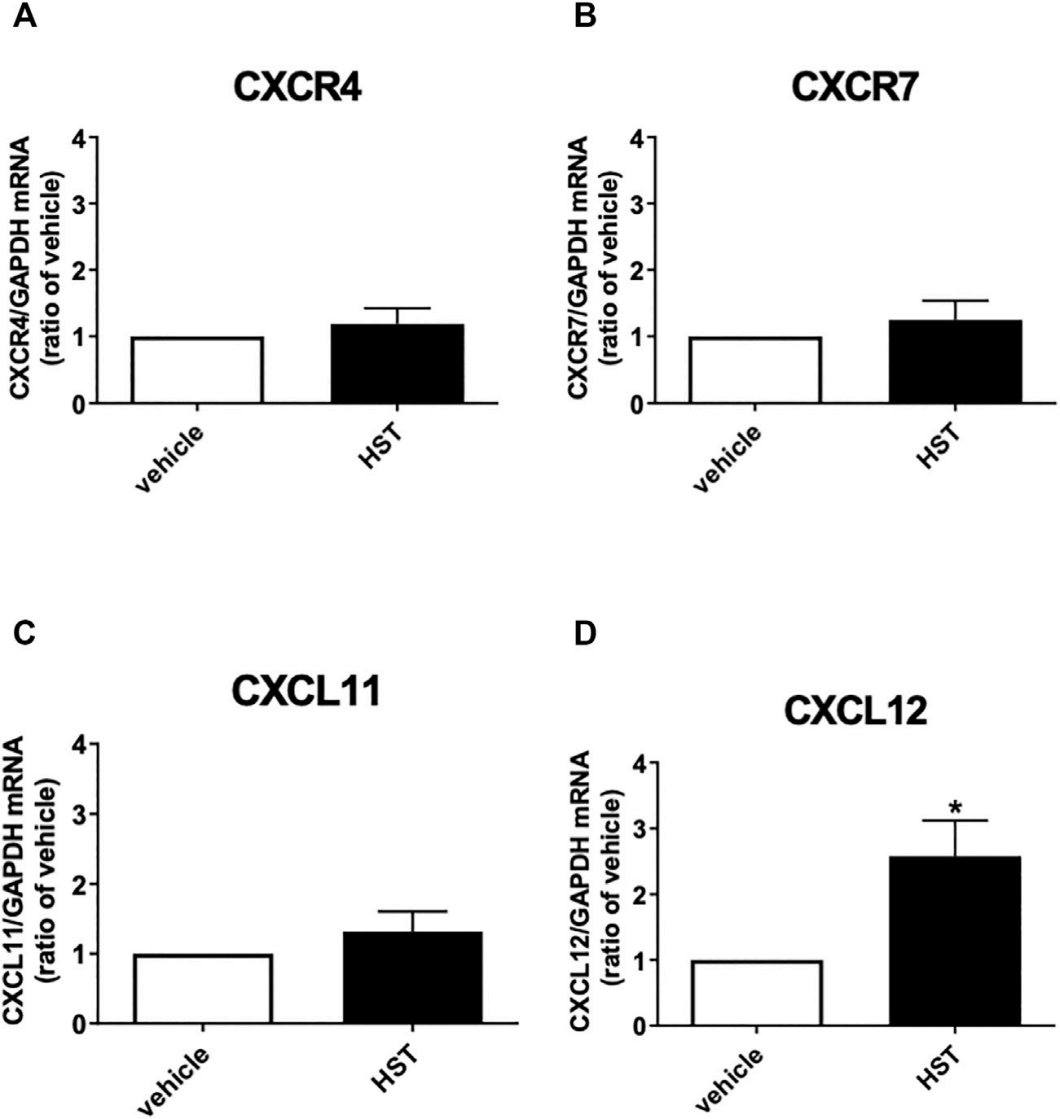
Effect of Hangeshashinto (HST) on the mRNA expression of CXCR4, CXCR7, CXCL11, or CXCL12 in human oral keratinocytes (HOKs). HOKs were scratched and treated with vehicle or HST for 48 h mRNA expression was determined by RT-qPCR. **(A)** CXCR4 (*n* = 5), **(B)** CXCR7 (*n* = 6), **(C)** CXCL11 (*n* = 5), or **(D)** CXCL12 (*n* = 6). Data are presented as the mean ± SEM (bars). * indicates *p* < 0.05, compared with vehicle; unpaired *t*-test.

All data are presented as the mean ± standard error of the mean (SEM) for at least three independent experiments. Statistical analysis was performed using one-way analysis of variance (ANOVA), followed by the Bonferroni’s multiple comparisons test (Figures 1, 2, 5) or unpaired *t*-test (Figures 2, 4), using GraphPad Prism version 8 software (GraphPad Software, La Jolla, CA, United States). A probability value (*p*) < 0.05 was considered statistically significant.

## 3 Results

### 3.1 HST Enhanced Scratch-Induced HOK Migration via MAPKs and CXCR4

We previously reported that treatment with 1–100 μg/ml HST enhanced scratch-induced wound healing in dose- and time-dependent manners, which involved HOK migration (Miyano et al., 2020). As shown in Figure 1, treatment with HST for 72 h significantly induced HOK migration. Conversely, this effect was significantly suppressed by treatment with a CXCR4 inhibitor (BDPA-Zn, 3 µM), an ERK inhibitor (U0126, 10 µM), a JNK inhibitor (JNK inhibitor II, 1 µM), and a p38 inhibitor (SB202190, 10 µM). Compared with vehicle treatment alone, treatment with each inhibitor did not significantly affect HOK migration (Figure 1).

### 3.2 HST Upregulated CXCL12, But Not CXCR4, CXCR7, Nor CXCL11 in HOKs

To clarify the molecular mechanism responsible for HST-induced HOK migration, we first investigated the effects of HST on the expression of endogenous CXCR4 agonists (CXCL11 and CXCL12) and the receptors CXCR4 and CXCR7 in HOKs. As shown in Figure 2, treatment with HST for 48 h significantly increased mRNA expression of CXCL12, but not that of CXCR4, CXCR7, nor CXCL11, compared with vehicle treatment.

### 3.3 HST Upregulated CXCL12 via ERK Activation in HOKs

**FIGURE 3:**
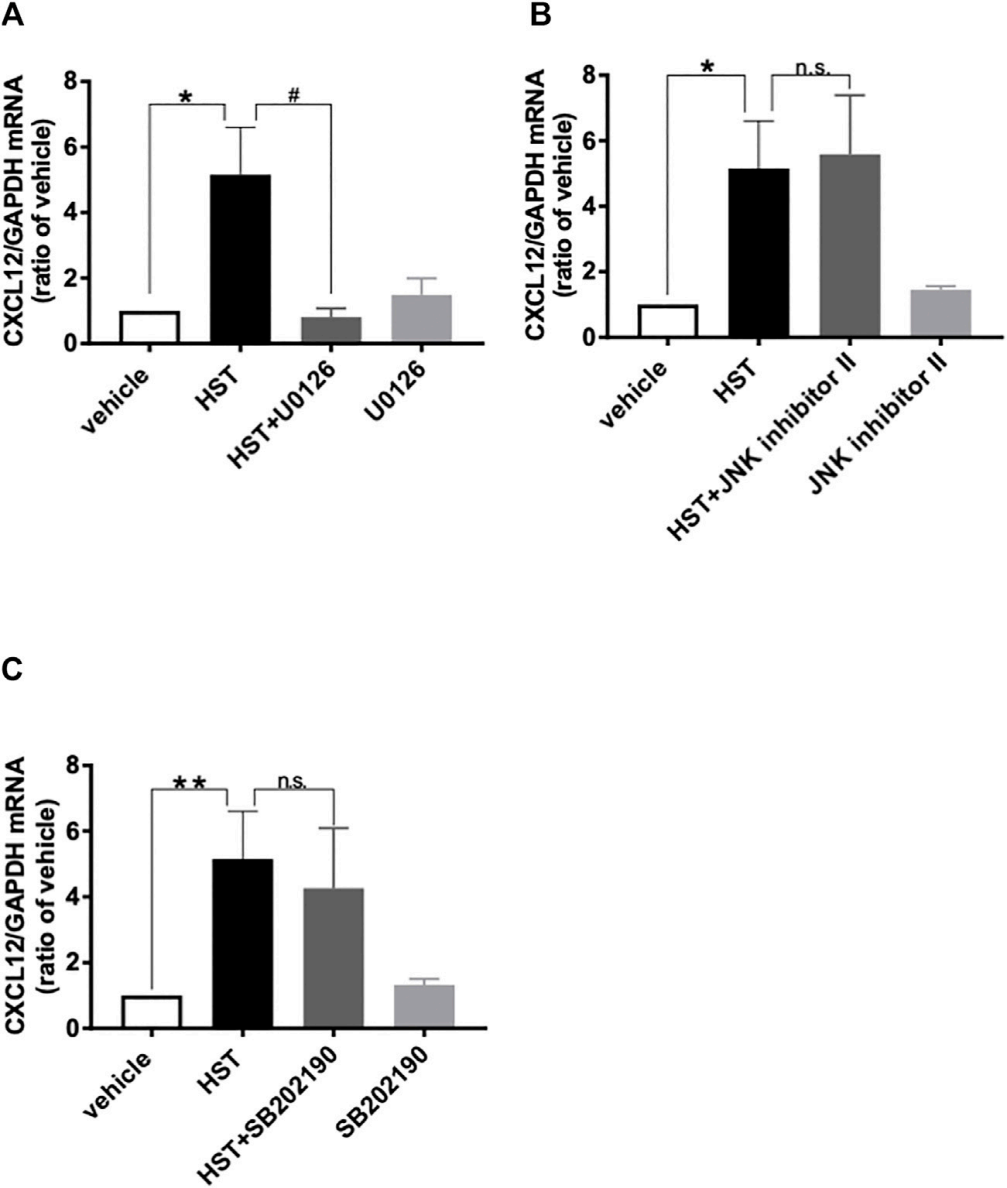
Involvement of MAPKs in Hangeshashinto (HST)-induced expression of CXCL12 mRNA in human oral keratinocytes (HOKs). HOKs were scratched and co-treated with HST and inhibitors of **(A)** ERK (U0126; *n* = 3–5), **(B)** JNK (JNK inhibitor II; *n* = 3–5) or **(C)** p38 (SB202190; *n* = 3–8) for 48 h. mRNA expression was determined by RT-qPCR. Data are expressed as the mean ± SEM (bars). *, ** indicates *p* < 0.05, *p* < 0.01 compared with vehicle, respectively; # indicates *p* < 0.05 compared with HST alone. Bonferroni’s comparison test following ANOVA **(A–C)**. n.s. indicates not significant **(B,C)**.

**FIGURE 4:**
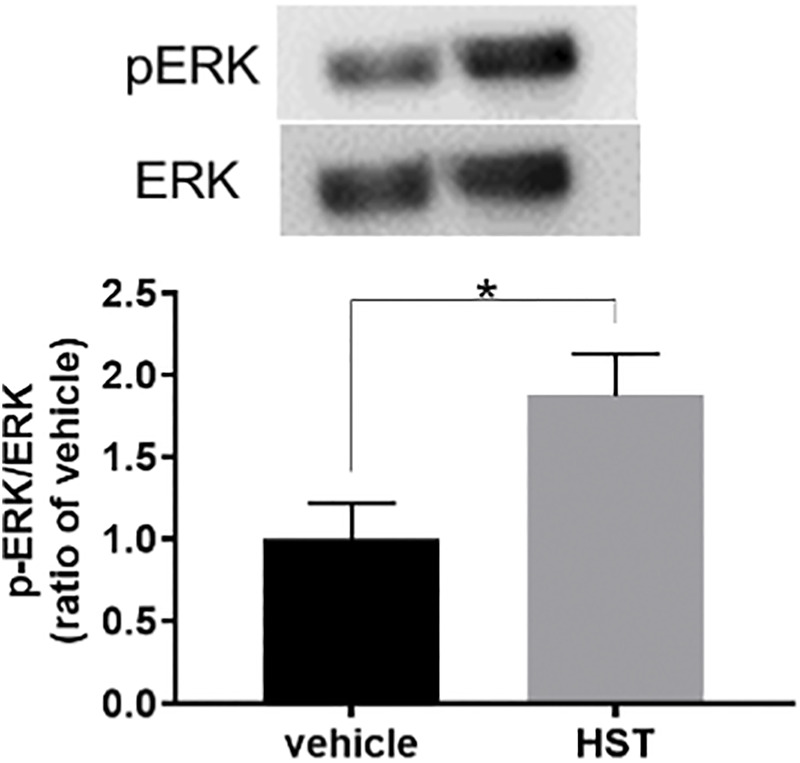
Effect of Hangeshashinto (HST) on ERK phosphorylation in human oral keratinocytes (HOKs). HOKs were scratched and treated with vehicle or HST for 48 h. ERK expression was calculated using the ratio of the pERK-specific band density/ERK-specific band density determined by western blotting (*n* = 3). Data are expressed as the mean ± SEM. (bars). * indicates *p* < 0.05 compared with vehicle. Unpaired *t*-test.

To elucidate the involvement of MAPKs in HST-induced upregulation of CXCL12, we examined the effects of MAPK inhibitors on HST-induced upregulation of CXCL12 in HOKs. The ERK inhibitor (U0126, 10 µM) completely suppressed HST-induced CXCL12 mRNA expression (Figure 3A), but this phenomenon was not observed when HOKs were treated with the JNK inhibitor (JNK inhibitor II, 1 μM, Figure 3B) nor with the p38 inhibitor (SB202190, 10 μM, Figure 3C). We then examined whether HST affected phosphorylation of ERK in HOKs using western blotting. The results indicated that HST treatment significantly increased ERK phosphorylation in HOKs compared with vehicle treatment (Figure 4).

### 3.4 10-gingerol Upregulated CXCL12 in HOKs

**FIGURE 5:**
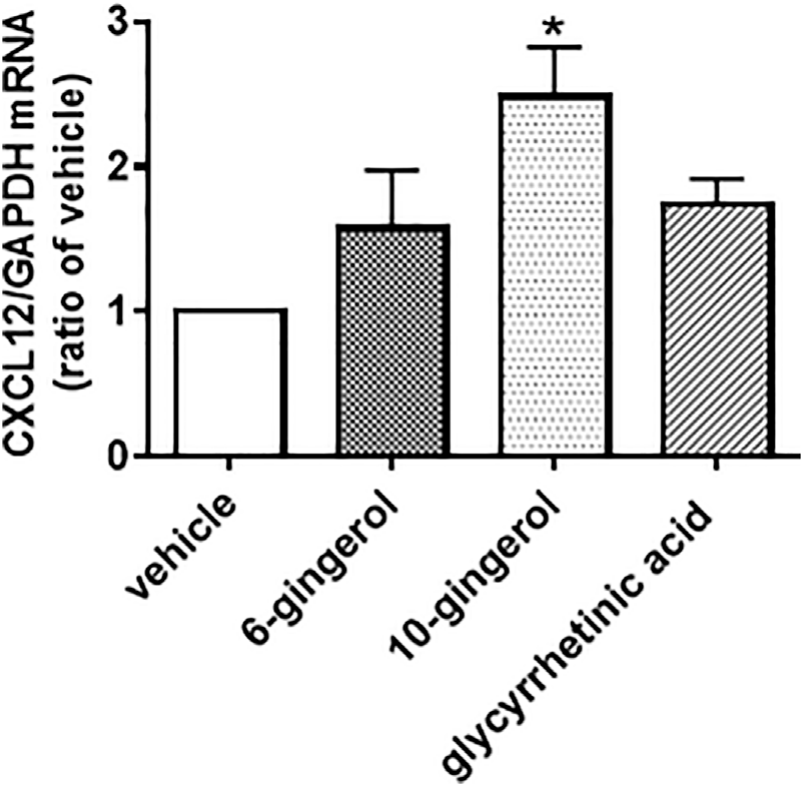
Effect of components of Hangeshashinto (HST) on the expression of CXCL12 mRNA in human oral keratinocytes (HOKs). HOKs were scratched and treated with vehicle, 6-shogaol (1 µM), 10-gingerol (10 µM), or glycyrrhetinic acid (10 µM) for 48 h. mRNA expression was determined by RT-qPCR. Data are expressed as the mean ± SEM. (bars). * indicates *p* < 0.05 compared with vehicle. Bonferroni’s comparison test following ANOVA.

Our previous study revealed that 6-shogaol, 10-gingerol and glycyrrhetinic acid, which are the typical components of HST, enhanced the scratch-induced HOK migration (Miyano et al., 2020). We examined the effects of these components on the level of CXCL12 mRNA expression in HOKs. The doses of these compounds used in the present study were determined according to our previous study (Miyano et al., 2020), which have highest efficacy in the scratch-induced migration. As shown in Figure 5, 10-gingerol (10 µM) significantly induced CXCL12 expression, compared with vehicle. Although 6-shogaol (1 µM) and glycyrrhetinic acid (10 µM) slightly increase CXCL12 expression, these responses were not significant.

## 4 Discussion

In this study, we revealed for the first time that treatment with 100 μg/ml HST activated ERK and upregulated CXCL12 in HOKs, which subsequently caused their migration. OUM treatment in clinical practice involves dissolving HST in hot water to a final concentration 50 mg/ml, followed by mouth washing (Matsuda et al., 2015). Our previous study determined that the doses of HST-derived compounds required for effective HST-induced HOK migration were higher than the concentrations of HST-derived compounds found in patient plasma (Miyano et al., 2020). However, the effective doses in the HST-induced HOK migration assay were lower than those measured in the HST solution used in clinical practice (Matsuda et al., 2015; Miyano et al., 2020). Taken together, these findings suggest that HST-induced CXCR12 expression via ERK activation is evoked by direct action of HST on OUM, not following absorption in the blood.

**FIGURE 6:**
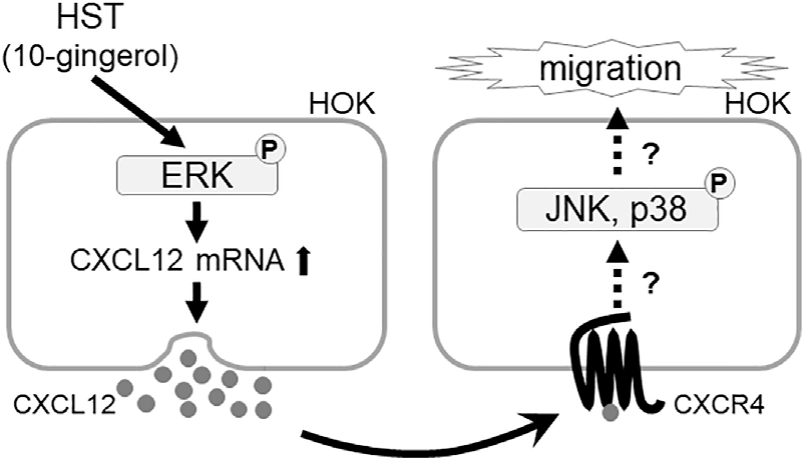
Schematic diagram showing the mechanism of the Japanese herbal medicine HST inducing oral keratinocyte migration by mediating the expression of CXCL12 through the activation of ERK. HST induces ERK phosphorylation and upregulates CXCL12, which activates its receptor CXCR4, and induces cell migration.

Many studies have reported that MAPK, CXCR, and CXCL play important roles in cell migration (Kukreja et al., 2005; Huang et al., 2009; Yuan et al., 2013; Cui et al., 2016). Concurring with the results of previous studies, our results demonstrated that ERK, JNK, p38, and CXCR4 inhibitors significantly suppressed HST-induced HOK migration (Figure 1). Shi et al. determined that CXCL12 was upregulated via ERK activation (Shi et al., 2013). In addition, some reports have shown that cell migration induced by the CXCL12/CXCR4 axis is the result of ERK, JNK, and p38 activation (Sun et al., 2002; Kukreja et al., 2005; Huang et al., 2009; Yuan et al., 2013; Cui et al., 2016). We found that HST-induced CXCL12 expression was involved in the activation of ERK, but not that of JNK and p38 (Figure 3). HST increased the phosphorylation level of ERK in HOKs (Figure 4). Taken together, these data suggest that HST induces ERK phosphorylation and upregulates CXCL12, which activates its receptor CXCR4, and consequently induces cell migration through phosphorylation of JNK and p38 (Figure 6). However, further studies are needed to clarify the effects of CXCL12 on HOK migration via JNK and/or p38.

Our previous study revealed that Scutellaria root (baicalein), processed ginger (6-shogaol, 8-shogaol, 10-shogaol, 6-gingerol, 8-gingerol, and 10-gingerol), and Glycyrrhiza (glycyrrhetinic acid) were the active constituents among the seven botanical drugs comprising HST, suggesting that these ingredients could cooperatively enhance scratch-induced HOK migration (Miyano et al., 2020). Some studies have reported that baicalein activates JNK and/or p38 (Chao et al., 2007; Su et al., 2018), while 6-shogaol and 10-gingerol activate ERK, JNK, and p38 (Kim et al., 2009; Kim et al., 2015; Ryu and Chung, 2015). These studies suggest that 6-shogaol and 10-gingerol induce ERK phosphorylation, resulting in production of CXCL12 in HOKs. In fact, our present study revealed that 10-gingerol significantly induced mRNA expression of CXCL12 in HOKs (Figure 5). Taken together, these data suggest 10-gingerol induced CXCL12 expression via activation of ERK in HOKs. Further investigation is warranted to elucidate which ingredients including 10-gingerol activate ERK, JNK, and p38.

Our previous study indicated that HST enhances tissue repair using animal models of chemotherapy-induced OUM (Miyano et al., 2020). The migration of keratinocytes is the basis for re-epithelialization during wound healing (Castellano-Pellicena and Thornton, 2020). In our wound healing assay using HOKs, the scratched HOKs produced inflammatory mediators such as PGE2 (data not shown), which were elicited during chemotherapy and such inflammatory mediators induced OUM (Miyano et al., 2016). These data suggest that our cell culture model of HOK migration reflects one of mechanism of chemotherapy-induced OUM. However, further investigations are needed to reveal relationship between HOK migration induced by HST and this tissue repair using both *in vitro* and vivo assay.

In conclusion, the findings of the present study suggest that treatment with HST enhances tissue repair through oral keratinocyte migration likely induced by CXCR4 activation through upregulation of CXCL12 via activation of ERK. In addition, we identified 10-gingerol to induce CXCL12 expression in HOKs among components of HST. However, it is not clear whether 10-gingerol induces CXCL12 expression via ERK in HOKs. Further investigations using *in vivo* and *in vitro* assay are needed to reveal that 10-gingerol improves mucositis via the ERK-CXCL12-CXCR4 pathway. Nonetheless, this study provides scientific evidence supporting the use of HST in patients with cancer and comorbid OUM.

## Data Availability

The original contributions presented in the study are included in the article/Supplementary Material, further inquiries can be directed to the corresponding author.
