# Supplementary material for: The Japanese Herbal Medicine Hangeshashinto Induces Oral Keratinocyte Migration by Mediating the Expression of CXCL12 Through the Activation of Extracellular Signal-Regulated Kinase
Source: Front Pharmacol. 2022 Jan 18;12:695039. doi: 10.3389/fphar.2021.695039 (PMC8822321; doi:10.3389/fphar.2021.695039)
Supplement: Supplementary file 1 [file DataSheet1.PDF]

Supplementary Material

Name of Raw Material	Batch No. of Raw Material
	HST lot No. 2180014010
Pinellia tuber	37301120
Scutellaria root	37301090<br>37301100
Processed ginger	37300690<br>37300920
Glycyrrhizae Radix	37300530<br>37300540<br>37300560
Ziziphi Fructus	37300430<br>37301130
Ginseng Radix	37301050
Coptis rhizome	37301160<br>37301170

Supplementary Table 1. List of raw materials with batch number.

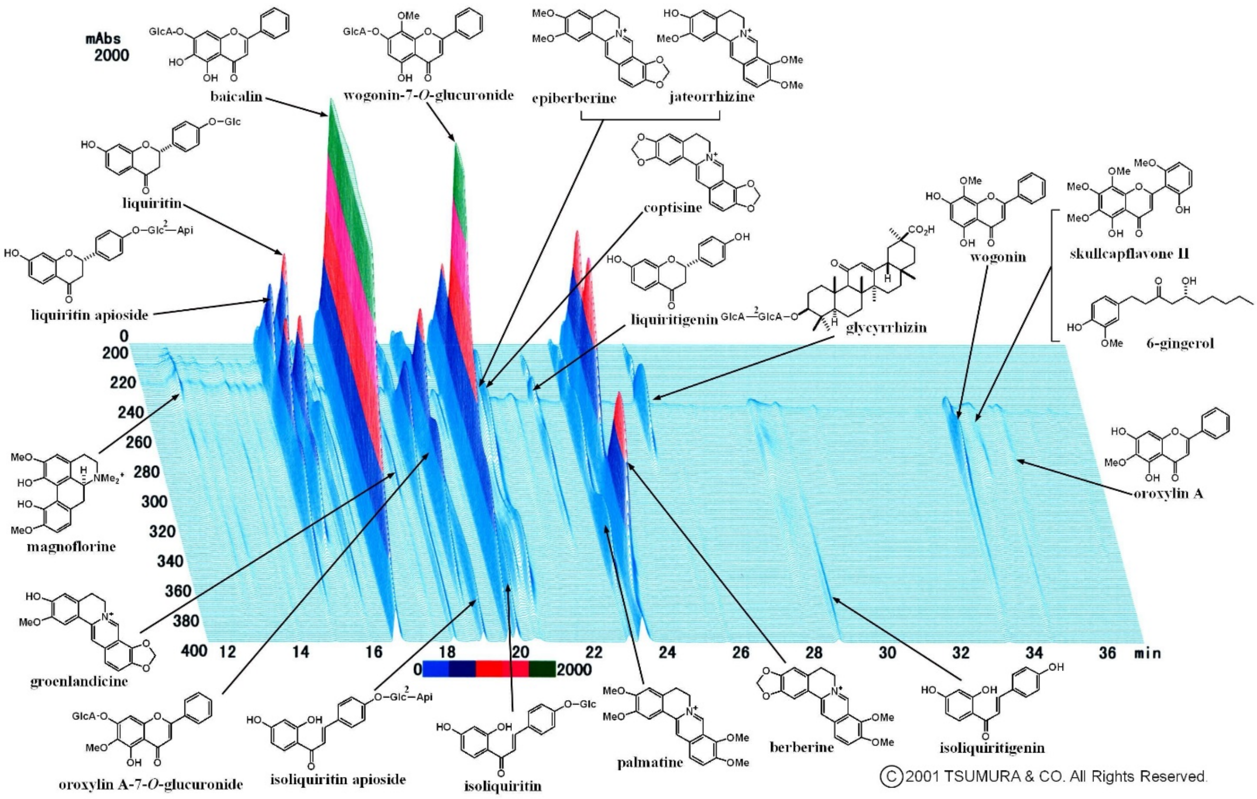

Supplementary Figure 1. The three-dimensional HPLC profiles of HST
